# The Value of Automated Diabetic Retinopathy Screening with the EyeArt System: A Study of More Than 100,000 Consecutive Encounters from People with Diabetes

**DOI:** 10.1089/dia.2019.0164

**Published:** 2019-10-21

**Authors:** Malavika Bhaskaranand, Chaithanya Ramachandra, Sandeep Bhat, Jorge Cuadros, Muneeswar G. Nittala, Srinivas R. Sadda, Kaushal Solanki

**Affiliations:** ^1^ Eyenuk, Inc., Los Angeles, California.; ^2^ EyePACS LLC, San Jose, California.; ^3^ Doheny Eye Institute, Los Angeles, California.

**Keywords:** Artificial intelligence, Automation, Diabetic retinopathy, Screening

## Abstract

***Background:*** Current manual diabetic retinopathy (DR) screening using eye care experts cannot scale to screen the growing population of diabetes patients who are at risk for vision loss. EyeArt system is an automated, cloud-based artificial intelligence (AI) eye screening technology designed to easily detect referral-warranted DR immediately through automated analysis of patient's retinal images.

***Methods:*** This retrospective study assessed the diagnostic efficacy of the EyeArt system v2.0 analyzing 850,908 fundus images from 101,710 consecutive patient visits, collected from 404 primary care clinics. Presence or absence of referral-warranted DR (more than mild nonproliferative DR [NPDR]) was automatically detected by the EyeArt system for each patient encounter, and its performance was compared against a clinical reference standard of quality-assured grading by rigorously trained certified ophthalmologists and optometrists.

***Results:*** Of the 101,710 visits, 75.7% were nonreferable, 19.3% were referable to an eye care specialist, and in 5.0%, the DR level was unknown as per the clinical reference standard. EyeArt screening had 91.3% (95% confidence interval [CI]: 90.9–91.7) sensitivity and 91.1% (95% CI: 90.9–91.3) specificity. For 5446 encounters with potentially treatable DR (more than moderate NPDR and/or diabetic macular edema), the system provided a positive “refer” output to 5363 encounters achieving sensitivity of 98.5%.

***Conclusions:*** This study captures variations in real-world clinical practice and shows that an AI DR screening system can be safe and effective in the real world. This study demonstrates the value of this easy-to-use, automated tool for endocrinologists, diabetologists, and general practitioners to address the growing need for DR screening and monitoring.

## Introduction

About 30.2 million adults 18 years of age or older, or 12.2% of all U.S. adults, had diabetes as of 2015.^1^ About 7.2 million of these adults were not aware that they had the disease or did not report that they had it.^2^ The global prevalence of diabetes among adults older than 18 rose from 4.7% in 1980 to 8.5% in 2014.^3^ The microvascular complications of diabetes are the leading cause of new-onset blindness and vision loss among working-age individuals in the developed world.^4^ The number of individuals affected by diabetic eye disease is rising. Between 2005 and 2050, the number of Americans 40 years of age or older with diabetic retinopathy (DR) is predicted to triple from 5.5 to 16 million people, and sight-threatening DR (STDR) will rise from 1.2 million in 2005 to 3.4 million in 2050.^5^

The American Diabetes Association (ADA) 2019 position statement recommends a comprehensive, dilated eye examination at the time of diagnosis of diabetes and every 1–2 years thereafter if no retinopathy is detected.^6^ If any level of DR is present, subsequent dilated retinal examination should be repeated at least annually.^6^ Typically a diabetologist or general physician will refer a patient with diabetes to an eye care specialist, with whom there may be a prolonged wait for an appointment.^5,7^ Screening and monitoring of DR are of vital importance, as early detection and treatment of DR have been shown to delay or prevent vision loss or reverse DR signs, and significantly slow DR progression.^4,5^

Despite the availability of evidence-based prevention and treatment protocols for DR, less than 62% of individuals with diabetes received recommended preventive care (DR screening) in 2015, leaving more than 35% at risk for vision loss or blindness.^1^ In addition, the currently used manual DR screening setups cannot scale up to effectively triage the ever-increasing population of people with diabetes at risk for vision loss, and the limited number of ophthalmologists, creating a large unmet need for screening.^8^

The authors believe that this need can only be met by a fully automated DR screening system (ADRSS) able to triage patients who require referral to an eye care specialist by analyzing color fundus images captured using a fundus camera.^9^ Such ADRSSs hold the potential to make the DR screening process more efficient, cost-effective, reproducible, and accessible. While there has been considerable interest in artificial intelligence (AI) for automated screening, adoption has been slow. Previous studies of AI-based automated DR screening have been on relatively small numbers of subjects in clinical trial settings that do not comprehensively capture variations encountered in real-world clinical practice.^10–12^ Broad user experience across diverse patient populations and settings is needed to demonstrate that an ADRSS is safe and effective (with high sensitivity and specificity and high negative predictive value [NPV]), is robust to imaging conditions typically found in real-world screening setups, and can perform screening in accordance with internationally recognized standards, as done in clinical practice today. Such large real-world studies can help build trust in ADRSSs and increase comfort in its use and uptake into routine clinical practice.

The EyeArt system v2.0 (Eyenuk, Inc., Los Angeles, CA) is a computerized, cost-effective, cloud-based AI medical device that endocrinologists, diabetologists, and general practitioners can easily use to rapidly and accurately screen color fundus images for DR and generate a report within minutes, which indicates detection of referral-warranted DR. A “positive” test result would warrant immediately scheduling an appointment with an eye care specialist and a “negative” test results warrants screening again in 9–12 months.^13,14^ In line with the American Academy of Ophthalmology's (AAO) preferred practice pattern guidelines, a patient is deemed referable if they have moderate nonproliferative diabetic retinopathy (NPDR), severe NPDR, proliferative DR, and/or clinically significant macular edema (CSME) surrogate markers in either eye,^15^ that is, hard exudates within one optic-disk diameter of the macula. A patient is deemed nonreferable if there are mild or no signs of DR and no CSME surrogate markers in both eyes.^15^

The EyeArt system's innovative AI technology combines novel morphological image analysis with state-of-the-art deep-learning techniques to create an ADRSS engineered for large-scale deployment in the cloud. Approximately 100,000 patients can be screened in less than 45 h, whereas screening of retinal images by a human grader can only evaluate 8–12 patients per hour. The EyeArt system is available 24/7 and supports retinal image processing in large batches. In a previous study with 40,542 images collected from 5084 diabetic patient encounters, an earlier version of the EyeArt system (v1.2) achieved 90% sensitivity at 63.2% specificity.^14^ The present REtrospective Validation of Eyeart in the REal world (REVERE) study assessed the diagnostic efficacy of the EyeArt system v2.0 screening in 107,001 consecutive diabetic patient visits from the EyePACS telescreening program. In this REVERE 100k study article, we improve on previous study and present data on the use of automated screening with mydriatic (with dilation) and nonmydriatic (without dilation) imaging protocols. It also demonstrates the value of this automated tool to endocrinologists, diabetologists, and general practitioners to address the growing need for DR screening and monitoring, remove the burden of DR screening from eye care specialists, and potentially preserve the vision of many people with diabetes worldwide.

## Methods

### Study design

This retrospective noninterventional REVERE study was performed on a cohort of 107,001 unselected consecutive diabetes patient visits/encounters and included 850,908 fundus images collected at 404 primary care clinics in the EyePACS DR telescreening program between January 2014 and September 2015. Each encounter is defined as a set of images captured during one patient visit with a predetermined fundus photography protocol. EyePACS is a retinal telescreening system that sends electronic digital images collected in one setting to a provider in another location.^16,17^ Ocular telemedicine and telehealth systems like EyePACS have the potential to decrease vision loss from DR.^18^ For this study, de-identified retinal images from EyePACS, including 54,481 nonmydriatic (53.6%), 46,580 mydriatic (45.8%), and 649 with unknown dilation status (0.6%) encounters, were uploaded to the EyeArt system cloud in a secure, encrypted transfer compliant with the Health Insurance Portability and Accountability Act (HIPAA). The validation data were independent from the data used to design and develop the EyeArt system.

The EyePACS imaging protocol requires three photographic fields: primary—centered between the disk and macula, nasal—centered on the disk, and temporal—centered about one disk diameter temporal to macula. In addition, an external eye image per eye is required. More than 90% of the encounters contained eight images each. This study employed the EyeArt system v2.0. The core DR analysis algorithms for the EyeArt system v1.2 using traditional machine learning have been described previously. These algorithms allowed for greater than 90% sensitivity, but specificity was a modest 60%–70%.^14^ The EyeArt system v2.0 combined these traditional features with those derived from multiple convolutional neural networks.^19^

The EyeArt system screening performance in identifying patients with at least one retinal image showing signs of referable DR was evaluated against the screening recommendations made by certified graders providing the clinical reference standard “ground truth” grading. EyePACS expert graders were certified, trained optometrists or ophthalmologists. They received rigorous training and certification through the EyePACS Retinopathy Grading System. The graders provided the international clinical diabetic retinopathy severity level and noted the presence/absence of CSME surrogate markers for determining the clinical management of patients. This quality-controlled, standardized grading by certified and trained experts is used as a reference standard to evaluate the EyeArt system DR screening recommendations.^17^ No patient was excluded from the study because of the number of images in the encounter or the format and/or resolution of the images.

Statistical analyses in this study were conducted using Python v2.7 and NumPy v1.9.2. The sensitivity (true positive [TP] rate) and specificity (true negative [TN] rate) of the EyeArt system algorithm for detecting DR and diagnosing referable/STDR were calculated by generating 2 × 2 tables, taking the human grader's assessment as the reference standard. Additional metrics of the EyeArt system screening performance to detect referable DR and sensitivity to detect potentially treatable DR included the positive predictive value (PPV), defined as the probability of presence of disease, given a positive test result, and the NPV, defined as the probability of the absence of disease, given a negative test result. Ninety-five percentage confidence intervals (95% CIs) were calculated for sensitivity, specificity, treatable DR sensitivity, NPV, and PPV.^20^ A receiver operating characteristic (ROC) curve was created by plotting the TP rate against the false positive (FP) rate at various threshold settings, and the area under the curve was calculated to determine how easy it is to distinguish between DR and non-DR groups. The EyeArt system performance was also stratified by dilation status (mydriatic vs. nonmydriatic encounters) of the patients.

## Results

**FIG. 1.**
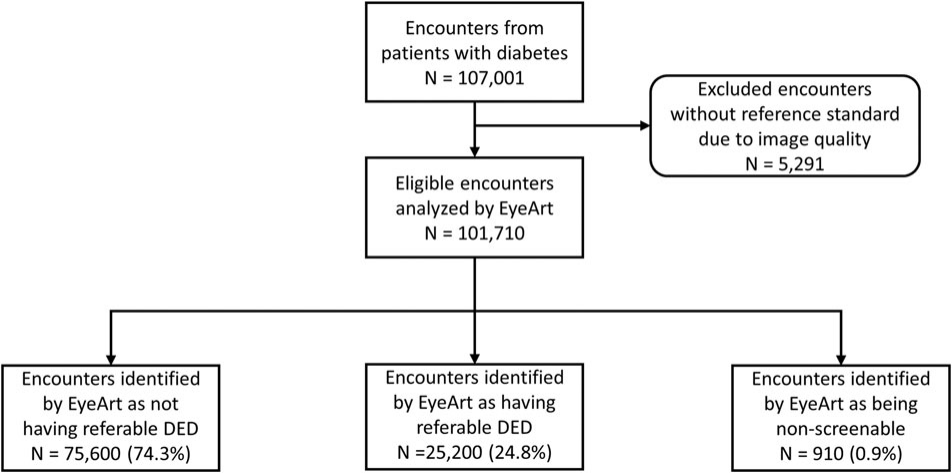
Distribution of patients with diabetes in REVERE 100k study. Around 4.9% of the encounters were excluded because of the lack of reference standard due to inadequate image quality. EyeArt system analyzed the remaining encounters, resulting in the following distribution: nonreferable DED (74.3%), referable DED (24.8%), and nonscreenable (0.9%). Images deemed “nonscreenable” by the EyeArt system were considered referable and included in the data analyses. DED, diabetic eye disease; REVERE, REtrospective Validation of Eyeart in the REal world.

Automated analysis and secure reporting for the entire cohort of 107,001 consecutive encounters with 850,908 color fundus images were completed in less than 45 h. Of these, 5291 encounters (4.9%) had missing DR and CSME levels as per the reference standard and thus were excluded from this analysis. The EyeArt system performance was evaluated on the remaining 101,710 encounters. Among the 101,710 encounters, only 910 encounters (0.9%) were flagged as being nonscreenable by the EyeArt system. These were given a “refer” output report and were included in the performance evaluation as having referable DR detected by the EyeArt system (Fig. 1).

The distribution of DR severity in the EyePACS screening population is summarized in Table 1. A patient was deemed nonreferable if there were mild or no signs of DR and no CSME surrogate markers in both eyes as per grading by EyePACS experts. Of the 101,710 encounters, 75.7% were nonreferable, 19.3% were referable to an eye care specialist, and DR level was unknown in 5.0% of the population.

**Table 1.** Diabetic Retinopathy Determination in the 107,001 Encounters

*DR severity (ICDR scale)*	*Episodes,* n *(%)*	*Referable/Nonreferable DED*
No apparent DR (0)	72,189 (67.5)	Nonreferable
Mild NPDR (1)	8816 (8.2)	
Moderate NPDR (2)	15,177 (14.2)	Referable
Severe DR (3)	2625 (2.5)	
Proliferative DR (4)	2819 (2.6)	
DR level unknown (−1)	5373 (5.0)	With CSME: Considered referable Missing CSME grade: Excluded

CSME, clinically significant macular edema; DED, diabetic eye disease; DR, diabetic retinopathy; ICDR, international clinical diabetic retinopathy; NPDR, nonproliferative diabetic retinopathy.

Of the 101,710 encounters, the EyeArt system reported 18,917 that were TP, 73,797 that were TN, 7193 that were FP, and 1803 that were false negative (FN). The output is presented in Table 2. The EyeArt system screening sensitivity was 91.3% (95% CI: 90.9–91.7) and specificity was 91.1% (95% CI: 90.9–91.3). The PPV was 72.5% (95% CI: 71.9–73.0), and the NPV was 97.6% (95% CI: 97.5–97.7). Of the 1803 FN encounters, 95.4% did not meet general treatment criteria because they had moderate NPDR. The area under the ROC curve (AUROC) was 0.965 (95% CI: 0.963–0.966).

**Table 2.** Metrics Used to Evaluate the EyeArt System

	*Condition of diabetes patient*	
	*Referable DR*	*Nonreferable DR*
EyeArt system test result		
Positive	TP = 18,917	FP = 7193
Negative	FN = 1803	TN = 73,797
Sensitivity = TP/(TP + FN) = 91.3%		
Specificity = TN/(TN + FP) = 91.1%		
Accuracy = (TN + TP)/(TN + TP + FN + FP) = 91.2%		
PPV = TP/(TP + FP) = 72.5%		
NPV = TN(TN + FN) = 97.6%		

FN, false negative; FP, false positive; NPV, negative predictive value; PPV, positive predictive value; TN, true negative; TP, true positive.

A subset of 192 patient encounters of the 101,710 encounters was randomly selected to be re-graded by an expert at the Doheny Eye Institute (DEI). On this subset, the agreement between EyePACS graders and DEI expert was substantial when considering encounters that are nonscreenable to be positive for referable DR by EyePACS graders and DEI expert (kappa of 0.69), and when ignoring encounters that are nonscreenable as per either EyePACS graders or DEI expert (kappa of 0.76). In this subset, when compared to grading by EyePACS graders, the EyeArt system had a 95.1% sensitivity for referable DR (within the encounters that were positive for referable DR as per the DEI expert) and a 98.3% specificity (within the encounters that were negative for referable DR as per the DEI expert).

In addition, the EyeArt system gave “positive” for referral-warranted DR to 5363 out of 5446 encounters with potentially treatable DR (severe or proliferative). The sensitivity for providing “positive” for referral-warranted DR for encounters with potentially treatable DR therefore was 98.5%. For potentially treatable DR, the fraction of FN in the entire cohort was 0.08% (83/101,710). The NPV for potentially treatable DR was 99.9%. A review of the 83 FN encounters with potentially treatable DR by an expert at the DEI detected only 16 encounters with potentially treatable DR. After adjudication by the DEI expert, the EyeArt system missed only 16 cases with potentially treatable DR, which equates to less than 0.3% of the total 5379 encounters with potentially treatable DR and less than 0.02% of the total 101,710 encounters analyzed. There were 5721 encounters that were positive for CSME (grading based on surrogate markers) and the EyeArt system identified these encounters with a sensitivity of 97.1% (95% CI: 96.7–97.6).

**FIG. 2.**
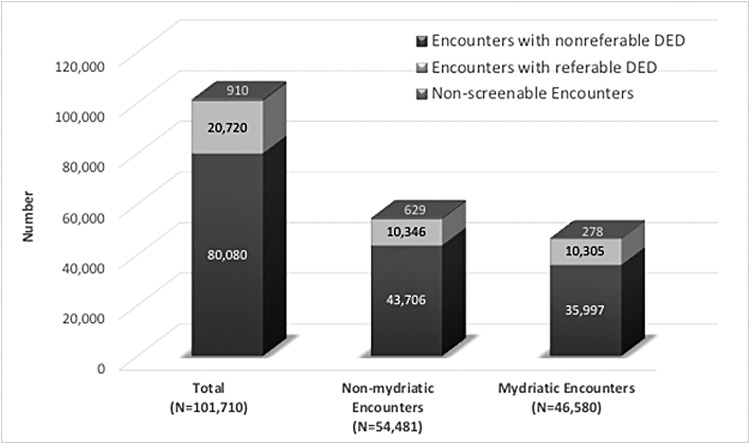
The number of nonreferable, referable, and nonscreenable encounters in the total population (*N* = 101,710), and as a function of dilation status in 53.6% of the encounters that were nonmydriatic and 45.8% of encounters that were mydriatic. The 0.6% of encounters whose dilation status was not known is not shown.

**FIG. 3.**
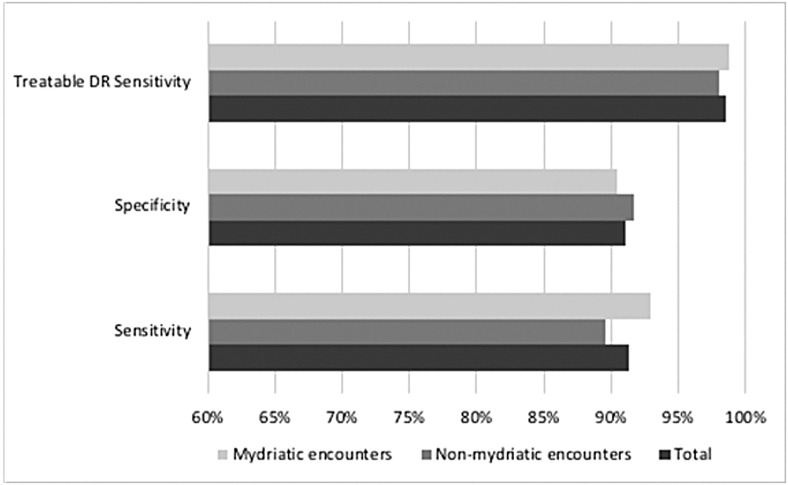
The sensitivity, specificity, and treatable DR sensitivity were determined for both mydriatic (*n* = 46,580) and nonmydriatic encounters (*n* = 54,481), and for the total population (*N* = 101,710) using the standard formulas shown in Table 2. DR, diabetic retinopathy.

The EyeArt system results for all encounters were stratified based on the dilation status of the encounter. The number and percentages of total nonmydriatic and mydriatic encounters were similar as were the numbers of encounters with referable DR as per the reference standard. The numbers and percentages of nonscreenable encounters were higher for nonmydriatic encounters. Dilation status was missing for 649 cases (0.6%) of the total encounters and 69 encounters with referable DR as per the reference standard (Fig. 2). The EyeArt system has high screening sensitivity and specificity on both mydriatic and nonmydriatic retinal images (Fig. 3). As shown in Table 3, sensitivity was 89.6% and 93.0% and specificity was 91.7% and 90.4% for nonmydriatic and mydriatic encounters, respectively. Treatable DR sensitivity was 98.0% and 98.8%, respectively, for nonmydriatic and mydriatic encounters captured after dilation.

**Table 3.** The EyeArt System Results for All Encounters and Results Stratified Based on the Dilation Status of the Encounter

	*Total*	*Nonmydriatic encounters*	*Mydriatic encounters*	*Dilation status missing*
Num. Encs. (% of total Encs.)	101,710 (100.0)	54,481 (53.6)	46,580 (45.8)	649 (0.6)
Sensitivity	91.3%	89.6%	93.0%	—
Specificity	91.1%	91.7%	90.4%	—
PPV	72.5%	71.7%	73.3%	—
NPV	97.6%	97.4%	97.9%	—
Treatable DR sensitivity	98.5%	98.0%	98.8%	—
AUROC	0.965	0.959	0.971	—
Num. Nonscreenable Encs. (% of Num. Encs.)	910 (0.9)	629 (1.2)	278 (0.6)	—

Number of samples *N* = 101,710. The nonscreenable encounters were considered referable and included in analysis.

AUROC, area under receiver operating characteristics curve; Encs., Encounters.

**FIG. 4.**
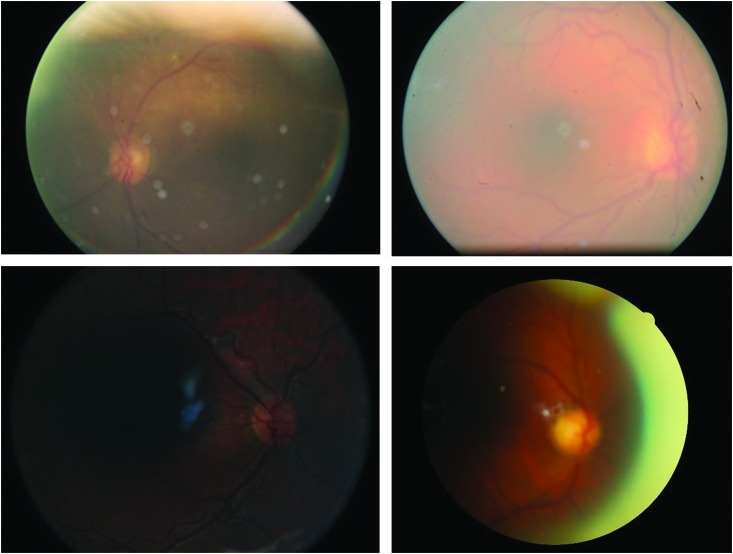
Example of images that are of poor image quality due to one or more of the following: insufficient focus, significant under or over exposed areas, presence of image artifacts like lens smudges, flares, or scratches. Inclusion of such images in an encounter may cause the EyeArt system to flag the encounter as nonscreenable. (Color images are available online.)

The EyeArt system needs at least one gradable quality image per eye (centered around the macula) to provide a screening output for the encounter. An encounter is flagged as nonscreenable if one or more of the encounter images are not gradable due to insufficient focus, under or over exposed images, insufficient retinal field of coverage, large lens smudges, scratches, or other such imaging artifacts (Fig. 4). In this study, among the 910 encounters (0.9%) flagged as being nonscreenable by the EyeArt system, 342 had potentially treatable DR, of which 198 encounters had quality marked as “insufficient for full interpretation” by EyePACS graders. Nonscreenable encounters flagged by the EyeArt system are treated as being positive for referral-warranted DR and therefore, the safety of these patients is not affected due to the encounter being nonscreenable.

## Discussion

A majority of DR screening programs use nonmydriatic retinal photography with a digital fundus camera to acquire color images of the retina that are then manually examined for the presence of DR pathology.^21,22^ Some DR screening programs may also choose to dilate patients (for mydriatic retinal photography) if gradable quality fundus images cannot be acquired otherwise. The use of manual graders to screen fundus images requires specific training, is time-consuming, is open to variability in interpretation, and cannot keep up with the large and rapidly growing diabetic population, thus leaving a significant number of patients vulnerable to vision loss or blindness.^14^ Automated screening methods based on algorithms capable of detecting lesions associated with DR is an efficient, cost-effective, reproducible, and accessible process^23^ that can help overcome barriers to screening, thereby allowing patients to meet ADA recommendations for DR screening.^6^

The EyeArt system is an AI-based software system for detecting DR.^24^ Its algorithm is machine learning based and trained on large numbers of cases that have contributed to refine the algorithm.^11^ The real-world study on over 100,000 consecutive visits of patients with diabetes reported in this article demonstrated that automated DR screening using the EyeArt system v2.0 achieves high screening sensitivity (91.3%) and specificity (91.1%). Since the study cohort contains a very large number of consecutive patient encounters obtained in real-world clinical settings from 404 primary care clinics in the EyePACS DR telescreening network, the authors believe that it captures the variation in patient population (age, gender, ethnicity, and duration of diabetes), camera types, and image quality seen in a general DR screening population. A separate independent study of the EyeArt system on over 20,000 consecutive patient encounters showed that the sensitivity and specificity were not affected by patient ethnicity, gender, or camera type.^25^ Furthermore, sensitivity and specificity are higher than others previously reported, moderately large studies that evaluated more than 10,000 patients. In one study of 15,000 patients, the sensitivity and specificity were 66.4% and 72.8%, respectively.^26^ In another with 16,670 people with diabetes, sensitivity was 90%, but specificity was only 47.7% using the EyeCheck algorithm and 43.6% using the Challenge2009 algorithm.^27^ These sensitivity and specificity statistics are important, because there is evidence that screening combined with appropriate management can prevent up to 95% of cases of vision loss and blindness.^28^

This REVERE 100k study is important because it provides clinical validation, which is essential to evaluate the safety and effectiveness of ADRSSs. Four crucial factors that determine if a clinical validation study is generalizable to the intended user and patient population are met in this study: (i) Study size: the number of subjects should be large to include rare cases and achieve statistical and clinical significance. (ii) Unbiased selection of subjects: images from consecutive subject visits from multiple primary care sites should be used to ensure that the test subjects are representative of the intended patient population. (iii) Quality of reference standard: the reference standard used for evaluation should be standardized, that is, performed as a centralized assessment by ophthalmologists and optometrists trained and certified for grading color fundus images.^29^ (iv) Independence of testing: test images should be completely independent of those used to train the automated system.

The size of this study and the use of consecutive patient encounters allow it to represent geographic, demographic, and photographic variations encountered in real-world screening populations. A key strength of the REVERE 100k study design was that it was based on a cohort comprising consecutive cases, including all images captured for each case rather than only macula-centered images, as in previous studies.^11^ The real-world nature and size of the cohort have allowed for the inclusion of rare DR cases based on a wide array of scenarios, such as number and location of lesions and disease progression.

This study provides a follow-up on a previous health technology assessment completed at Moorfields Eye Hospital NHS Foundation Trust study using the EyeArt system v1.0 that included analysis of 20,258 consecutive patient visits and reported a sensitivity of 93.8% for referable retinopathy, and was found to be effective with respect to the number of false-positive results compared to human graders. In addition, the automated approach was considered a cost-effective alternative to manual grading.^25,30^

Other studies have shown that the EyeArt system performs well in screening for DR.^14,24^ An earlier version of the EyeArt system software (v1.2) demonstrated 90% sensitivity at 63.2% specificity on a dataset of 40,542 images from 5084 patient encounters.^14^ In a recent study of retinal images from 296 patients, which were captured using a portable smartphone-based imaging device, the EyeArt system provided 95.8% sensitivity and 80.2% specificity for detecting any DR and 99.1% sensitivity and 80.4% specificity for detecting STDR.^24^ Further clinical validation of the sensitivity and specificity of the EyeArt system v2.0 automatic DR screening system to detect referable DR has been achieved against reading center grading of the gold standard Early Treatment Diabetic Retinopathy Study 7-field stereoscopic fundus images (*N* = 755) in simulated screening populations.^31^

DR screening using trained graders is time-consuming and places a heavy burden on health care time and financial resources.^11,24^ This contributes to low screening rates, and thousands of cases of preventable blindness are not caught in a timely manner. ADRSSs can make point-of-care DR screening possible and more accessible at primary care and diabetes care locations, and thus help reduce vision loss due to DR and improve access to eye care professionals by increasing the necessary referrals (patients requiring eye care treatment and management for DR) and reducing their DR screening load. An independent health technology assessment of a previous version (v1.0) of the EyeArt system on 20,258 consecutive patient visits concluded that the EyeArt system saved costs compared with manual grading. Although a more detailed health-economic study may be necessary to quantify the cost savings of the newer EyeArt system v2.0, it can be stated with confidence that the improved specificity of the EyeArt system v2.0 compared to the previous version (v1.0) provides greater cost savings.

Rapid analysis of patient encounters in the cloud in a fraction of the time opens up the possibility of allowing clinicians who do not specialize in eye care to perform retinal image screens of their patients with diabetes, while the individual is already there for a general, health maintenance visit, obtain immediate results, and subsequently generate a referral to an eye care specialist for those patients who screen positive. Automated DR screening at the point of care can shift the screening process from the eye care specialist to the endocrinologist, diabetologist, or general practitioner. By increasing accessibility to screening, this novel AI technology can increase screening rates and referrals, thus reducing the incidence of vision loss caused by DR, while decreasing the need for ophthalmologists for routine screening visits.

Another important finding in this REVERE 100k study is that the EyeArt system is not influenced by mydriatic status: the EyeArt system has high sensitivity and high specificity for both nonmydriatic and mydriatic imaging. A screening program or clinic using the EyeArt system can choose whether or not to dilate their patients and which patients to dilate. This can be an important advantage when screening is done as part of general diabetes management at a nonvision specialist appointment.

One possible protocol to use the EyeArt system in a primary care or diabetology practice for screening for DR could be as follows:

- Confirm eligibility of the patient for screening (patient has a diagnosis of diabetes mellitus, does not have any persistent visual impairment, and is not contraindicated for fundus photography).
- Image the patient's eyes using a nonmydriatic fundus camera following an imaging protocol where at least one image centered around the macula is acquired for each eye.
- Submit images to the EyeArt system for analysis. If the result is positive for referable DR (more than mild NPDR, with or without the presence of CSME), the patient can be immediately referred to an eye care specialist. If the result is negative, the patient can return for their recommended annual DR screening next year.
- If encounter is flagged as nonscreenable due to poor quality images, imaging may be retried. If encounter continues to be nonscreenable after retrying, imaging may need to be done after dilation (if patient has no contraindications for dilation and dilation can be performed by the clinic staff) or the patient maybe directly referred to eye care specialist for follow-up.

There are three important limitations of this study: the REVERE 100k study design was retrospective, which may be perceived to suffer from selection or other bias compared to a prospective study design. For this reason, a large population (107,001) of consecutives cases from over 400 different primary care clinics was studied, which helps significantly reduce the selection bias.

The second limitation is the potential for misclassification by a single human grader and the lack of adjudicated multiple reader grading for each encounter, which is not possible in such a large study population. In smaller studies, multiple graders are used and any intragrader variability can be adjudicated, allowing the algorithm to perform better if subtle findings exist on images that might pose a challenge to a single grader. In this very large study, this limitation is managed by the quality control process and by the extensive training and certification process for EyePACS Retinopathy expert graders of color fundus images, which helps standardize the clinical reference standard. The quality control process includes an adjudicating consultant, usually a retinal specialist ophthalmologist, who is available to make decisions resolving issues of ambiguous or controversial interpretation. Adjudicating consultants may also perform quality control by reviewing a subsample of cases that were reviewed by other clinical consultants.^32^ Repeat grading by a retinal subspecialist who is an expert in the EyePACS grading protocol may also be included.^17^

A third limitation of this study is that data on the mean age of the patients or the mean duration of diabetes are unavailable to assess the performance of the EyeArt system, stratified on these factors. Media opacities and poor natural mydriasis may make it difficult to obtain good quality nonmydriatic images in older patients and those with long-term diabetes. DR screening programs may choose to dilate such patients for mydriatic photography, if gradable nonmydriatic images cannot be obtained. The EyeArt system has been shown to have high screening sensitivity and specificity on both mydriatic and nonmydriatic retinal images. Moreover, the use of a large study cohort of consecutive patient encounters captured in real-world clinical settings at 404 clinical sites improves the generalizability of the study results to real-world DR screening.

In summary, this large real-world REVERE study of over 100,000 consecutive patients from over 400 clinics demonstrated that automated screening using the EyeArt system was safe and effective, with high observed AUROC value and high observed sensitivity and specificity for detecting DR using both mydriatic and nonmydriatic imaging protocols. The study supports the application of AI with the EyeArt system for cost-effective mass DR screening with fewer human resources by cloud-based retinal image analysis. By eliminating a need for an eye care specialist for screening, the EyeArt system increases accessibility of DR screening, allowing timely management. Endocrinologists, diabetologists, and general practitioners can easily and accurately screen for DR within minutes during the patient's regular visit and determine whether the patient needs to be immediately referred to an eye care specialist. In daily practice, an easy-to-understand diagnostic report would be generated, allowing patients to be triaged at the point of care.

## References

[1] CDC Diabetes Report Card: 2017 www.cdc.gov/diabetes/library/reports/congress.html (accessed 1126, 2018)

[2] Center for Disease Control: National Diabetes Statistics Report, 2017 https://www.cdc.gov/diabetes/pdfs/data/statistics/national-diabetes-statistics-report.pdf (accessed 120, 2019)

[3] World Health Organization: Diabetes 2018 https://www.who.int/news-room/fact-sheets/detail/diabetes (accessed 120, 2019)

[4] National Eye Institute: Facts about diabetic eye disease. 2015 https://nei.nih.gov/health/diabetic/retinopathy (accessed 1125, 2018)

[5] Fox CR, Kronenberg K, Chu G, et al.: Increasing eye care screening & referral for people with diabetes via telehealth programs. US Department Health & Human Services http://dhhs.ne.gov/publichealth/Documents/Vision121010.pdf (accessed 1125, 2018)

[6] ADA Position Statement Standards of Medical Care in Diabetes: 2019 http://clinical.diabetesjournals.org/content/diaclin/early/2018/12/16/cd18-0105.full.pdf (accessed 120, 2019)

[7] Hartnett ME, Key IJ, Loyacano NM, et al.: Perceived barriers to diabetic eye care: qualitative study of patients and physicians. Arch Ophthalmol 2005;123:387–391 15767483 10.1001/archopht.123.3.387

[8] International Council of Ophthalmology: Number of Ophthalmologists in Practice and Training Worldwide. www.icoph.org/ophthalmologists-worldwide.html (accessed 120, 2019)

[9] Abràmoff MD, Niemeijer M: Mass screening of diabetic retinopathy using automated methods. In: Michelson G, ed. Teleophthalmology in Preventive Medicine. Berlin, Heidelberg: Springer Berlin Heidelberg, 2015, pp. 41–50

[10] Abramoff MD, Lavin PT, Birch M, et al.: Pivotal trial of an autonomous AI-based diagnostic system for detection of diabetic retinopathy in primary care offices. Digital Med 2018;1:39 10.1038/s41746-018-0040-6 PMC6550188 31304320

[11] Gulshan V, Peng L, Coram M, et al.: Development and validation of a deep learning algorithm for detection of diabetic retinopathy in retinal fundus photographs. JAMA 2016;316:2402–2410 27898976 10.1001/jama.2016.17216

[12] Van der Heijden AA, Abramoff MD, Verbraak F, et al.: Validation of automated screening for referable diabetic retinopathy with the IDx-DR device in the Hoorn Diabetes Care System. Acta Ophthalmol 2018;96:63–68 29178249 10.1111/aos.13613 PMC5814834

[13] Solanki K, Ramachandra C, Bhat S, et al.: EyeArt system: automated, high-throughput, image analysis for diabetic retinopathy screening. IOVS 2015;56 Abstract 1429

[14] Bhaskaranand M, Ramachandra C, Bhat S, et al.: Automated diabetic retinopathy screening and monitoring using retinal fundus image analysis. J Diabetes Sci Technol 2016;10:254–261 26888972 10.1177/1932296816628546 PMC4773978

[15] Diabetic Retinopathy Preferred Practice Pattern: 2017 https://www.aao.org/preferred-practice-pattern/diabetic-retinopathy-ppp-updated-2017 (accessed 120, 2019)

[16] EyePACS DR Screening: www.eyepacs.com/eyepacssystem (accessed 1125, 2018)

[17] Cuadros J, Bresnick G: EyePACS: an adaptable telemedicine system for diabetic retinopathy screening. J Diabetes Sci Technol 2009;3:509–516 20144289 10.1177/193229680900300315 PMC2769884

[18] Li HK, Horton M, Bursell S-E, et al.: Telehealth practice recommendations for diabetic retinopathy, second edition. Telemed J E Health 2011;17:814–837 21970573 10.1089/tmj.2011.0075 PMC6469533

[19] Simonyan K, Zisserman A: Very Deep Convolutional Networks for Large-Scale Image Recognition. ArXiv 2014 http://arxiv.org/abs/1409.1556

[20] Altman D, Machin D, Bryant T, Gardner M, eds.: Statistics with Confidence: Confidence Intervals and Statistical Guidelines, 2nd ed. Great Britain: BMJ Books, 2000

[21] Pieczynski J, Grzybowski A: Review of diabetic retinopathy screening methods and programmes adopted in different parts of the world. Eur Ophthal Rev 2015;9:49–55

[22] Chakrabarti R, Harper CA, Keefe JE: Diabetic retinopathy management guidelines. Expert Rev Ophthalmol 2012;75:417–439

[23] Nørgaard MF, Grauslund J: Automated screening for diabetic retinopathy: a systematic review. Ophthalmic Res 2018;60:9–17 29339646 10.1159/000486284

[24] Rajalakshmi R, Subashini R, Anjana RM, Mohan V: Automated diabetic retinopathy detection in smartphone-based fundus photography using artificial intelligence. Eye 2018;32:1138–1144 29520050 10.1038/s41433-018-0064-9 PMC5997766

[25] Tufail A, Rudisill C, Egan C, et al.: Automated diabetic retinopathy image assessment software: diagnostic accuracy and cost-effectiveness compared with human graders. Ophthalmology 2017;124:343–351 28024825 10.1016/j.ophtha.2016.11.014

[26] Walton IV OB, Garoon RB, Weng CY, et al.: Evaluation of automated teleretinal screening program for diabetic retinopathy. JAMA Ophthalmol 2016;134:204–209 26720694 10.1001/jamaophthalmol.2015.5083

[27] Abràmoff MD, Reinhardt JM, Russell SR, et al.: Automated early detection of diabetic retinopathy. Ophthalmology 2010;117:1147–1154 20399502 10.1016/j.ophtha.2010.03.046 PMC2881172

[28] Abràmoff MD, Niemeijer M, Suttorp-Schulten MS, et al.: Evaluation of a system for automatic detection of diabetic retinopathy from color fundus photographs in a large population of patients with diabetes. Diabetes Care 2008;31:193–198 18024852 10.2337/dc08-0952 PMC2494619

[29] Wong TY, Bressler NM: Artificial intelligence with deep learning technology looks into diabetic retinopathy screening. JAMA 2016;316:2366–2367 27898977 10.1001/jama.2016.17563

[30] Tufail A, Kapetanakis VV, Salas-Vega S, et al.: An observational study to assess if automated diabetic retinopathy image assessment software can replace one or more steps of manual imaging grading and to determine their cost-effectiveness. Health Technol Assess 2016;20:1–104 10.3310/hta20920 PMC5204130 27981917

[31] Solanki K, Bhaskaranand M, Bhat S, et al.: Comprehensive clinical validation study of an automated DR screening system against 7-field ETDRS stereoscopic reference standard. Am Acad Ophthalmol 2016 Abstract 30049096

[32] Cuadros J, Martin C: Diabetic retinopathy screening practice guide. In: Yogesan K, Goldschmidt L, Cuadros J, eds. Digital Teleretinal Screening. Berlin, Heidelberg: Springer Berlin Heidelberg, 2012, pp. 11–30

